# Rheology of Polymer Processing in Spain (1995–2020)

**DOI:** 10.3390/polym13142314

**Published:** 2021-07-14

**Authors:** Leire Sangroniz, Mercedes Fernández, Pedro Partal, Antxon Santamaria

**Affiliations:** 1 POLYMAT and Department of Polymers and Advanced Materials: Physics, Chemistry and Technology, Faculty of Chemistry, University of the Basque Country UPV/EHU, Paseo Manuel de Lardizábal, 3, 20018 Donostia-San Sebastián, Spain; leire.sangroniz@ehu.es (L.S.); mercedes.fernandez@ehu.es (M.F.); 2 Pro2TecS—Chemical Process and Product Technology Research Centre, Department of Chemical Engineering, ETSI, Universidad de Huelva, 21071 Huelva, Spain; partal@uhu.es

**Keywords:** rheology, processing, thermoplastics, thermosets, biopolymers, composites, adhesives, bitumen

## Abstract

The contribution of Spanish scientists to the rheology involved in polymer processing during the last 25 years is investigated. It is shown that the performed research covers, at different levels, all industrial polymeric materials: thermoplastics, thermosets, adhesives, biopolymers, composites and nanocomposites, and polymer modified bitumen. Therefore, the rheological behaviour of these materials in processing methods such as extrusion, injection moulding, additive manufacturing, and others is discussed, based on the literature results. A detailed view of the most outstanding achievements, based on the rheological criteria of the authors, is offered.

## 1. Introduction and Some Remarks on the Rheology in Spain

The first steps in the field of rheology in Spain were carried out in the Fat Research Institute of Seville and in the Polymer Science and Technology Institute (ICTP) (formerly *Instituto de Plásticos y Caucho*) (Madrid), both of which have belonged to the Spanish National Research Council (CSIC) since 1947. Professors Martinez Moreno and Gómez Herrera initiated the studies of the rheology and interfacial aspects of water and tensioactive systems [1] at the Fat Research Institute, whereas Professor Martín Guzmán set up the first investigations on polymer rheology at the ICTP-CSIC. Two facts contributed decisively to the spreading of the rheology in Spain: The collaboration of Professor Gómez Herrera with the University of Sevilla and, on the other hand, the successive professor positions reached by Gonzalo Martín Guzmán at the Polytechnic University of Catalunya (UPC) and The University of the Basque Country (UPV-EHU).

As far as we are aware, Martín Guzmán and Gómez Fatou were the first Spanish researchers to publish a paper on rheology, in particular about the rheological behaviour of poly(methacrylate solutions) [2]. 

Considerably later than the Society of Rheology (1929), the British Rheology Society (1940), and the French Rheology Group (Groupe Français de Rhéologie) (1964), the Spanish Rheology Group (Grupo Español de Reología, GER) was created in 1983, under the auspices of the Spanish Royal Society of Chemistry and Spanish Royal Society of Physics. Professor Alemán Vega of ICTP-CSIC was elected as the first president of the GER in a meeting attended by 15 members of different universities, research centres, and industrial companies. Professors Críspulo Gallegos of the University of Sevilla (1993–2006) and Antxon Santamaria of the University of the Basque Country (2006–2015) succeed Professor Alemán Vega. Currently, the GER is led by Professor Antonio Guerrero of the University of Sevilla.

In the years since, the Spanish Rheology Group has gained an increasing influence among European rheologists, publishing scientific papers and organizing international meetings, such as the Fourth European Rheology Conference (Sevilla 1994), French Iberian Rheology Conference (Biarritz 1998), Eurorheo 2002 Joint Meeting of British, Italian, Portuguese, and Spanish Societies (Torremolinos 2002), and the XVIth International Congress on Rheology (Lisbon 2012, in collaboration with Portuguese and Slovenian Societies). The international activities of the GER were reflected in the appointment of Críspulo Gallego to the presidency of the European Society of Rheology (2009). 

In the past decades, there has been a close collaboration between the Spanish Rheology Group and the Portuguese Society of Rheology (SPR). Every two years, both institutions organize a joint meeting under the name Ibereo. Seven Ibereo conferences have been celebrated to date; the last in 2019 in Oporto. Both societies are in charge of the organization of the next Annual European Rheology Conference (AERC) to be held in Sevilla in April 2022.

**Figure 1:**
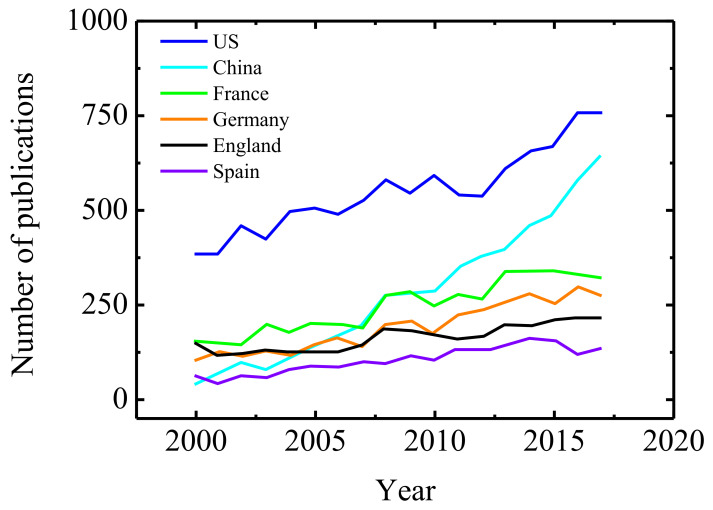
Number of publications on rheology for US, China, France, Germany, England, and Spain from 2000 to 2017. Taken from reference [3].

An article of Professor Antonio Guerrero in the bulletin of GER [3] offers an analysis of the papers on rheology published in journals of the Citation Index. We remark that the majority of the articles refers to polymers. Figure 1, which is taken from this reference, shows the evolution in different countries between 2000 and 2017. 

In this manuscript we focus on the rheology of polymer processing in Spain between 1995 and 2020. In terms of the number of papers, this represents approximately the half of the rheology of polymers in general, which covers works on characterization, macromolecular dynamics, and theory and simulation. 

In the field of polymer science and technology, the word *processing* stands for the process or method to fabricate any object from raw material. In a wider sense, *processing* can also be used as a stage to accomplish the final aim of a polymeric compound, as in the case of adhesives, coatings, and asphalt binders.

Depending on the physicochemical characteristics of each polymer, and on the features of the sample to be elaborated, different processing methods can be used, such as extrusion and injection moulding, additive manufacturing, etc.

The manuscript is divided in six sections, each one corresponding to the different families of industrial polymers: Thermoplastics, thermosets, adhesives, biopolymers, composites, and polymer modified bitumens. 

Indeed, the work is not free of a certain subjectivity, as we make a personal selection of what we consider the most outstanding outcomes. 

## 2. Thermoplastic Polymers

Rheology and thermoplastic polymers have been closely linked since their respective beginnings in the late twenties of the last century. Processing thermoplastics is a challenge that has always required the help of flow behaviour studies. Most of the research on polymer rheology has focused on industrial processing of thermoplastics and, on the other hand, it can be said that rheology advancement owes very much to the peculiar viscoelastic behaviour of polymer melts.

The most employed and investigated polymer processing methods involve shear flows, as is the case of extrusion moulding, injection moulding, compression moulding, calendaring, and extrusion based additive manufacturing (EAM). In the case of compression moulding, the produced squeezing flow is actually a shear flow. One of the historical highlights of rheology is the discovery of the dependency of viscosity on shear rate, i.e., the so-called *non-Newtonian viscosity.* During the flow developed under industrial processing conditions, the effect of shear rate on viscosity can be even more crucial than the effect of temperature and pressure. Indeed, the ranges of shear rates vary considerably with the contemplated industrial processing procedures. In Table 1, the shear rate intervals that correspond to each processing method are depicted.

Fortunately, molten polymers show a non-Newtonian viscosity with a shear thinning behaviour, which signifies that viscosity decreases as shear rate is enhanced. This gives rise to low viscosities for high-speed processes such as injection moulding, resulting in considerable reduction in energy consumption, not sufficiently recognized by ecological organizations. In view of this reality, it becomes clear that obtaining the viscosity curve (i.e., viscosity versus shear rate) of each polymer melt at the adequate temperature becomes a peremptory necessity for processing.

There are also relevant processing methods that involve extensional or stretching flows, as is the case of melt spinning, extrusion blowing, foaming, and calendaring. Certainly, research of extensional flows related to polymer processing is much less abundant than that of shear flows.

In Spain, as in other industrial countries, the number of articles regarding the rheology of thermoplastics processing is much higher than that of thermosets. Considerable research has been carried out, especially regarding rheology linked to extrusion moulding, and the collaboration between academia and industrial companies, such as REPSOL and ERCROS, must be pointed out. In Table 2, the number of papers published in the last 25 years by universities, research centres, and industries is shown. These numbers reflect, in some cases, collaborations between two Spanish institutions, so repeated articles may appear on counting. 

### 2.1. Extrusion Moulding

Data of the viscosity as a function of shear rate, which gives rise to the so-called viscosity curve, constitute the nucleus of the most important contribution of rheology to extrusion and injection moulding. Viscosity curves at different temperatures frame the extrusion conditions for each polymer sample, marking two crucial issues: energy consumption and surface quality of the moulded part. As a rule of thumb, the sample which offers the lowest viscosity should be selected, but the effect of temperature and shear rate must be taken into account. Reducing the viscosity at the expense of increasing temperature may be a bad choice, considering the balance of mechanical and thermal energy consumption. On the other hand, knowing the viscosities at the shear rates estimated for each processing method is essential, as having the lowest viscosity at shear rates in the range 10–100 s^−1^ does not necessarily signify the smallest viscosity during extrusion moulding (100–1000 s^−1^, Table 1), as the viscosity curves of two samples can cross. 

In the last 25 years, a number of Spanish papers [4,5,6,7,8,9,10,11,12,13,14,15,16,17,18,19,20,21,22,23,24,25,26,27,28,29,30,31,32,33,34,35,36] have dealt with viscosity results of polymer melts related to their aptness for extrusion moulding. The article of Vega et al. [7], the result of the collaboration between the University of the Basque Country and REPSOL, is the most cited among the Spanish papers properly focused on the rheology applied to polymer processing, according to the Web of Science.

**Figure 2:**
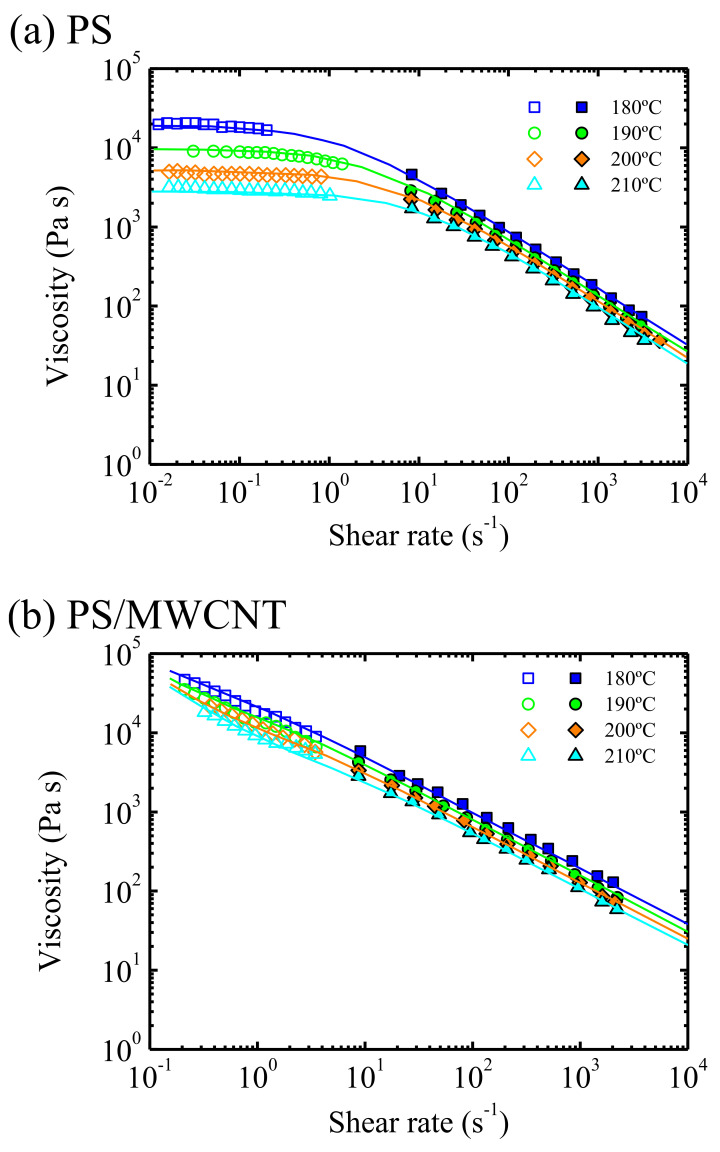

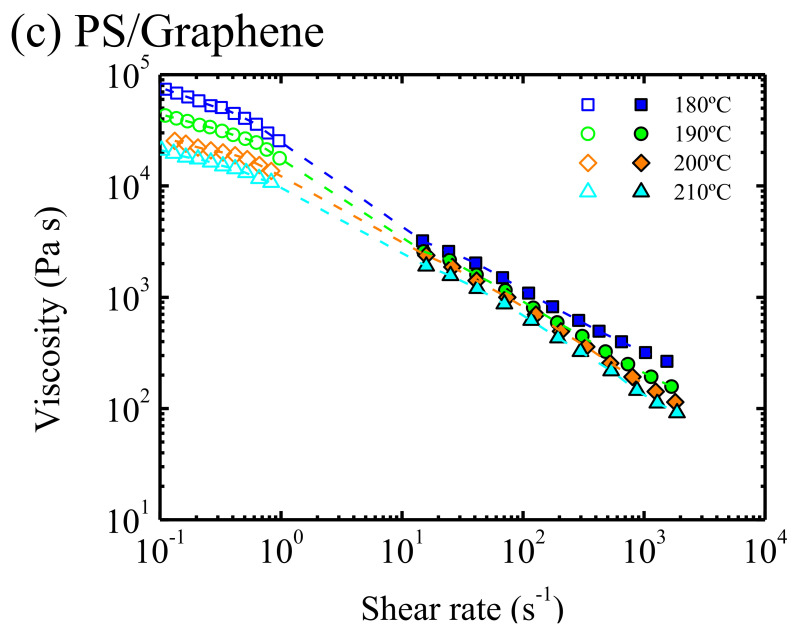
Viscosity as a function of shear rate for (**a**) PS, (**b**) PS/MWCNT, and (**c**) PS/PS/graphene. Open symbols correspond to data obtained with plate–plate geometry, whereas filled symbols correspond to data obtained by capillary extrusion rheometer [30]. The lines correspond to a modified Carreau Yasuda paper, see reference [30]; for Figure 2c, the discontinuous lines are to guide the eye. Reprinted with permission from [30]. Copyright 2016 The Society of Rheology.

Figure 2 is an example of the effect of shear rate and temperature on the viscosity of a polystyrene sample, obtained by two rheological techniques: plate-plate geometry and capillary extrusion rheometry [30]. A shear rate range of almost six decades is covered, but the values above 100 s^−1^ are the most relevant from the extrusion moulding point of view. Notwithstanding, the Newtonian (shear rate independent) viscosity obtained at the lowest shear rates is also very useful, as its value can be correlated with the molecular weight of the sample, as is seen, for instance, in a paper of Vega et al. [27].

**Figure 3:**
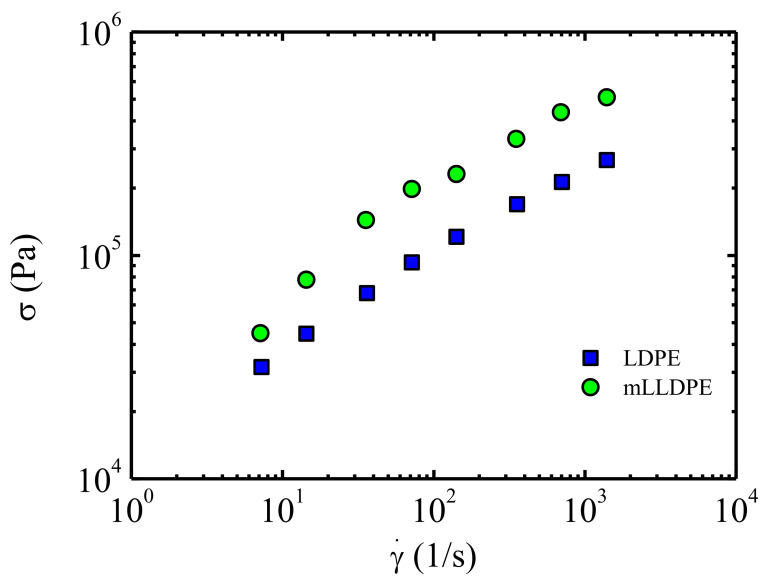
Flow curves of mLLPE and LDPE obtained by extrusion flow at 190 °C (“m” stands for metallocene-catalysed polyethylene; see text) [21] Reprinted with permission from [21]. Copyright 2005 Elsevier Ltd.

The research on metallocene-catalysed polyethylenes to produce tubes of a better quality by extrusion, started by the Spanish energy and petrochemical company REPSOL at the beginning of the nineties, opened the opportunity to work on the rheology of these new materials. The rheology groups of the University of the Basque Country (UPV/EHU) and the Institute for the Structure of Matter (CSIC, Madrid) were involved in this challenge. The desired improvement of the mechanical properties of extruded metallocene-catalysed polyethylenes is linked to a narrow molecular weight distribution, which typically results in higher viscosities above 100 s^−1^. As an example, the respective flow curves (shear stress versus shear rate) of a conventional polyethylene with wide molecular weight distribution and a metallocene-catalysed polyethylene with a narrow molecular weight distribution are compared in Figure 3, taken from the paper of Pérez et al. [21]. At a temperature of *T* = 190 °C the shear stresses required for the flow of the metallocene-catalysed polyethylene at the shear rates involved in tube extrusion (100 to 1000 s^−1^) double those required in the case of the conventional polyethylene, which results in a considerable increase in the energy consumption. 

**Figure 4:**
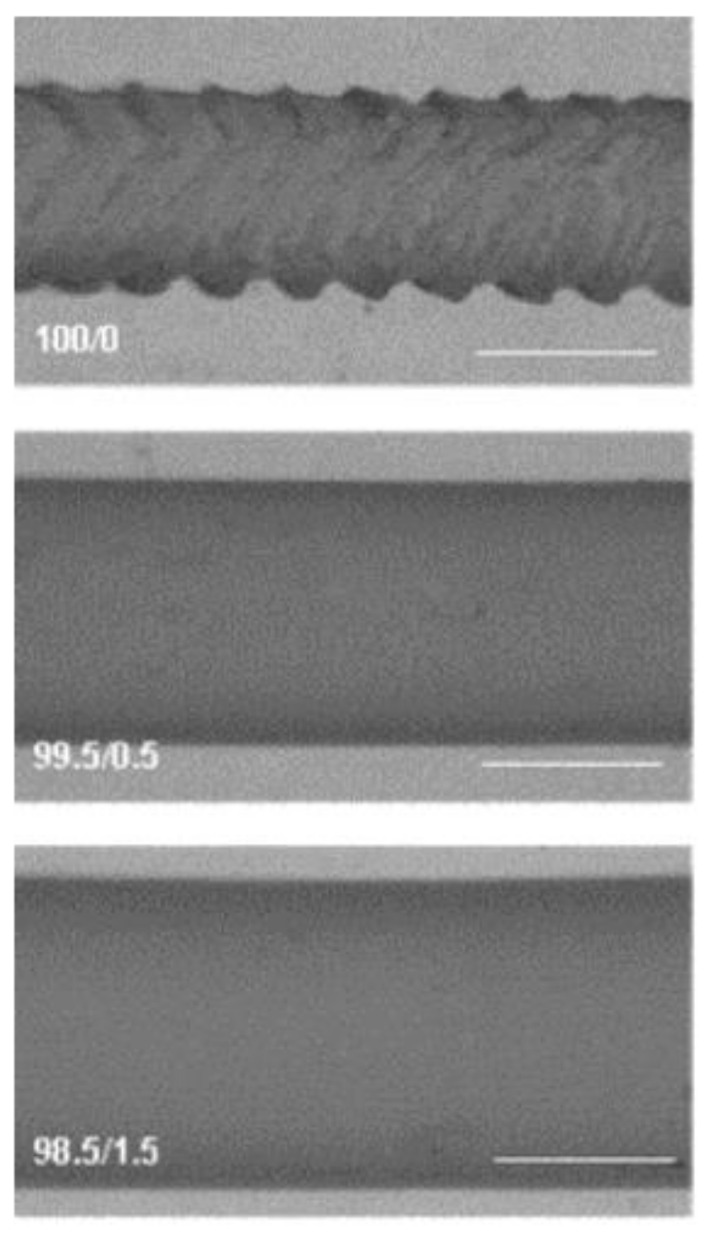
Microgaphs of metallocene polyethylenes and ultra-high molecular weight polyethylene blends of extrudates obtained at 169 °C. Adequate blend composition brings about smooth extrudates (bottom). The line in the picture corresponds to 1 mm [18] Reprinted with permission from [18]. Copyright 2004 American Chemical Society.

In the case of polyethylenes in general, shear stresses above around 2 × 10^5^ Pa give rise to the so-called *sharkskin* surface instability, which makes the polymer unable to elaborate tubes and other objects that require a smooth surface. Sharkskin in metallocene-catalysed polyethylenes is a real issue, as the shear stresses developed during extrusion in many cases exceed the critical value for the onset of this flow instability. Different strategies have been envisaged to avoid this problem, without increasing the extrusion temperature and keeping the good mechanical properties derived from the narrow molecular weight distribution, typical of metallocene-catalysed polyethylenes [9,12,13,16,17,21,25]. As an example, Aguilar et al. [18] show a route to eliminate sharkskin of metallocene-catalysed polyethylenes based on the addition of small amounts of high molecular weight samples. Figure 4, taken from this reference, shows the improvement of the surface smoothness, which is reached by applying the procedure described by the authors. 

While sharkskin surface instability is characteristic of the extrusion flow of polyethylenes, other commodity polymers, such as polystyrene and polypropylene, show a flow distortion, called “melt fracture”, which can also spoil material processing. Melt fracture is a consequence of the high elasticity (capacity to store energy) of some polymer melts, and usually takes place at low temperatures and high shear rates. In this case, the collaboration between polymer chemists and rheologists is conducted to postpone the onset of melt fracture to high enough shear rates, so processing is not affected. Spanish rheologists have worked on the melt fracture of polypropylene [22], poly(styrene-ethylene/buthylene-styrene) triblock copolymers [24], and poly(vinyl chloride) (PVC) [31]. As a matter of fact, research on the rheology of PVC processing has gained a certain reputation thanks to the collaboration between the Spanish company ERCROS and the Rheology group of the University of the Basque Country [14,15,19,32,33,34,35]. The most recent of these papers focuses on the rheology applied to additive manufacturing, which is treated in Section 2.3.

Out of the field of commodities, high performing thermotropic liquid crystal polymers, created as an alternative to liotropic liquid crystal polymers (e.g., Kevlar^®^), show peculiar and interesting rheological properties in the molten state. Extrusion and injection moulding of thermotropic liquid crystal polymers is conditioned by their special viscoelastic behaviour, which gives rise to negative normal forces and unexpected viscosity reductions. The latter are due to the orientation of the rod-like polymer chains, distinctive of thermotropics, during capillary or slit flow. Considering the little international scientific attention that the rheology of the processing of thermotropic liquid crystal polymers has attracted, the papers published by Spanish authors on thermotropic copolyesters can be considered as relevant [4,5,6,8,10]. 

The physico-chemical changes provoked by temperature, pressure, and shear stress during extrusion has been investigated by Spanish researchers. Quintana et al. [26] investigated the crystallization induced by shear flow in an amorphous branched poly(ethylene-co-1,4-cyclohexanedimethylene therephthalate) copolyester, monitoring the viscosity increase after an induction time. Additionally, a comparative study of flow-induced results in linear and branched polypropylenes was carried out by Vega et al. [23], deducing that higher level of molecular orientation was reached for branched sample. In turn, Sangroniz et al. [36] studied the effect of pressure and shear flow on the crystallinity of semi-crystalline polypropylene and polypropylene/polyethylene terephthalate blends, combining rheological and polymer characterization techniques. 

The alteration of the molecular parameters during processing, particularly in extrusion moulding, is the principal issue in the task of taking advance of recycled polymers. Rheological implications of recycling have been analysed by the Polymer Group of the University of Coruña [28] for the case of poly(ethylene terephthalate), which is one of the most recycled thermoplastics. On the other hand, the rheological and mechanical properties of a biopolymer, Poly(lactic acid), reprocessed several times in a extruder, was investigated by Peinado et al. of the University of Zaragoza [29].

### 2.2. Injection Moulding

Shaping polymer melts by injection moulding implies the development of higher shear rates than in any other processing method, as can be seen in Table 1. Additionally, the polymer melt may be submitted to pressures above 10^6^ kPa in the nozzle; measuring the rheological functions in such extreme conditions is not an easy task. Notwithstanding, data of the dependence of viscosity on shear rate, temperature, and pressure are essential to run thermoplastic injection moulding software, such as the currently well-known MOLDFLOW^R^ and CADMOULD^R^. Viscosity data under conditions close to those of injection moulding are quite frequently achieved in laboratories (e.g., POLYMAT in the Basque Country) which work for the industry. However, the number of scientific papers on the rheology of injection moulding is relatively low. 

The issue of the effect of pressure on viscosity of thermoplastics was considered by Spanish rheologists within the framework of the Virtual Injection Moulding VIM project of the European FP6-NMP Programme. A couple of papers have been published on the subject, proposing semi-empirical methods to determine the effect of pressure on viscosity of injection grade thermoplastics [37].

Probably the most relevant papers, at least in Spain, dedicated properly to the rheology of injection moulding, correspond to the work of Fernández et al. [38,39] at the University of Zaragoza. In these papers, a methodology to measure the rheological properties during the injection process is proposed, demonstrating the necessity of determining precisely the viscosity of the materials from the injection point of view. The work of Navarro et al. [40], from the Valencia Polythecnic University is also remarkable, as viscosity data of blends of virgin and recycled polypropylenes at shear rates above 10,000 s^−1^ are provided, and a predictive model, experimentally validated using filling tests in injection moulding machines, is proposed. For its part, three papers on the rheology of reprocessed polymers by injection moulding [41,42,43] have been published: two of them refer to pol(ethylene terephthalate) [41,42] and one to acrylonitrile-butadiene-styrene (ABS) copolymer [43]. 

### 2.3. Additive Manufacturing

Additive Manufacturing (AM) is the term used to name a set of novel technologies developed to make objects layer by layer under computer aided design software. Currently, AM is rapidly gaining ground as an excellent option to fabricate end-use products in automotive, aerospace, construction, and medicine. The so-called extrusion-based additive manufacturing (EAM) is the most used 3D printing process for thermoplastics. In many cases, a coil of continuous filament is used to feed the EAM device, but extrusion of pellets is also employed. 

The rheological implications of 3D printing in general, and EAM in particular, are evident, as a capillary flow is in the nucleus of the process. As remarked in Table 1, the range of shear rates involved in EAM of thermoplastics is wide (10 s^−1^ to 2000 s^−1^) as the printing velocity is related to the flow rate. Therefore, an analysis of the viscosity curves of the considered samples at the corresponding temperatures is necessary, to avoid a trial-and-error procedure. After extrusion, the molten polymer is deposited in the platform by gravity, creating the computer designed object layer by layer. This implies an elongational flow, which is not as relevant as the shear flow in the die, as the distance between the exit of the nozzle and the platform is very small.

Besides the analysis of the flow in the nozzle, the viscoelastic behaviour of the thermoplastic samples in the molten quiescent state is also crucial. The viscoelastic response is linked to polymer welding, and a good adhesion between layers is decisive to obtain a mechanically performing object. Very interesting rheological studies on the viscoelasticity linked to polymer chains inter-diffusion between layers have been published in recent years [44,45].

Although in Spain, as in the rest of the word, the number of papers on 3D printing is rapidly increasing to large numbers, research on the rheology of EAM of polymer thermoplastics is not yet extensive. A collaboration between the Valencia Polytechnic University and the Universidad Politécnica Salesiana (Ecuador) has led to a research on the EAM feasibility of poly(lactic acid)/halloysite nanotubes and poly(lactic acid)/carbon nanotubes nanocomposites, based on viscosity results obtained by capillary rheometry [46,47]. For their part, Ortega at al. of the University of Las Palmas de Gran Canaria [48] focus their work on a complete analysis of the viscosity in capillary flow, to provide a criterion to establish the best ranges of temperature and shear stress to obtain homogeneous filaments of ABS (acrylonitrile-butadiene-styrene), PLA (poly(lactic acid)), and PCL (polycaprolactone) used in EAM. 

**Figure 5:**
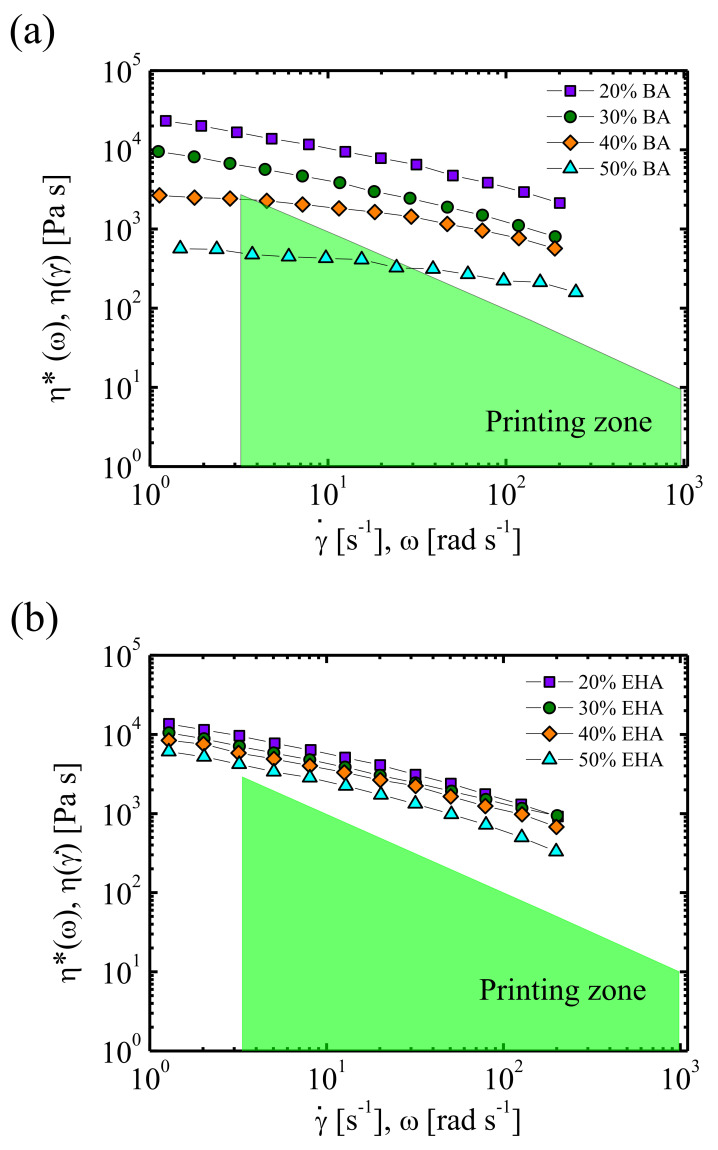
Complex viscosity versus frequency for PVC containing (**a**) poly(vinyl chloride-co-butyl acrylate) (PVC-BA) and (**b**) poly(vinyl chloride-co-2-ethylhexyl acrylate) (PVC-EHA) copolymers [32]. The printing zone is calculated modelling rheological conditions in the nozzle of the 3D printer Reprinted with permission from [32]. Copyright 2018 BME-PT.

The collaboration between the University of the Basque Country (UPV/EHU) and the Spanish company ERCROS has given rise to a series of papers on the rheological aspects of the printability of PVC and PVC copolymers [32,34,35]. A rheological analysis for adequate printing, considering the pressure and printing velocities ranges of the printing device, is shown in the paper of Calafell et al. [32]. In Figure 5, taken from this reference, the effect of the respective concentrations of butyl acrylate and ethyl-hexyl acrylate on the feasibility to 3D printing of PVC based copolymers is shown. The allowable printing conditions are delimited through viscosity measurements in the laboratory. Additionally, in the field of PVC derivates, Peñas et al. [34] have proposed a rheological model to face the issue of buckling of the filament during the feeding process of the EAM devices. 

Nowadays, the rapid development of thermoplastic biopolymers has its reflection in the research on the rheology of additive manufacturing at the UPV/EHU. Candal et al. [49] have investigated biodegradable poly(butylene succinate) (PBS) and poly(butylene succinate-*ran*-adipate) (PBSA) copolymers, observing that the viscosity results determine the good flow in the printer nozzle. Moreover, the relation between the values of the entanglements density, obtained by small amplitude oscillatory shear (SAOS) measurements, and the welding energy of the printed layers was demonstrated. It is also worth pointing out that the collaboration between the University of the Basque Country and the Brno Technical University (Czech Republic) has brought about a comparative study of the thermal and rheological features of poly(lactic acid) and poly(3-hydroxybutyrate) (PHB) based copolymers to deduce the suitability of each polymer to obtain scaffolds by EAM [50]. 

### 2.4. Film Blowing and Foaming

In shear flow the direction of the flow velocity and the direction of the velocity gradient are perpendicular. On the other hand, in a shear-free flow, the direction of the flow velocity and that of the velocity gradient coincide. In this kind of flow, an elongational or tensile stress and an elongational rate are defined, giving rise to an uniaxial elongational viscosity. The term elongational flow is in fact a synonym of shear-free flow. An ordinary example of uniaxial elongational flow is honey flowing down by gravity from a spoon: the velocity increases as the distance to the reference point increases. However, the presence of shear-free flows in nature is much more limited than that of shear flows. Shear flows are implied in crucial biological processes in life, such as blood flow in veins and arteries, capillary flows in plants, and many others.

Notwithstanding the fact that shear flows are involved in the majority of the polymer processing methods, there are very significant exceptions in which uniaxial and biaxial elongational flows play the principal role. Among these methods, we note the melt spinning of fibres, film blowing process, blow moulding, foaming, and calendaring. Rheology has reached a great progress on the study of the relation between molecular parameters of polymer chains and shear viscosity; thousands of scientific and technical papers have referred to this, bringing about one of the nuclei of rheology in general. However, this is not the case of elongational viscosity, which has received much less significant attention. 

The studies on the rheology of elongational flows in Spain are scarce. A collaboration between REPSOL and the University of the Basque Country for the study of polyethylene blends resulted in a paper on the correlation between elongational viscosity data (obtained through melt spinning) and blown film extrusion performance, showing the technical limitations caused by immiscibility [21]. In another paper [51], blown film properties of conventional and metallocene-catalysed polyethylenes were correlated with the melt elasticity procured from dynamic viscoelastic results. The results show that, although enhancing melt elasticity improves bubble stability, this provokes more haze and poorer dart impact properties. For its part, the collaboration between the Institute for the Structure of Matter CSIC (Madrid) and REPSOL led a paper on the melt strength of metallocene-catalysed polyethylenes immiscible blends in melt-spinning experiments [52]. Interestingly, from a physico-chemical point of view, a strong interaction between the high-molecular-weight linear fraction of the matrix and a fraction of molecules of the dispersed phase gives rise to a thick interface with its own viscoelastic properties, which determines melt-spinning results.

Biaxial elongational flows are involved in thermoplastics foaming processing methods, which encompass foam extrusion moulding and foam injection moulding. Among many other aspects of cellular polymers, the Cellular Materials Laboratory (CellMat) of the University of Valladolid has developed a notable research on the relation between uniaxial viscosity and the foamability of polyolefines [53,54,55]. In particular, an analysis of the strain hardening is achieved, concluding that an enhancement of strain hardening results in an increase in the polymer resistance to be stretched, which helps to avoid undesirable cellular degeneration. Three cases on the role played by strain hardening are considered by the authors: Strain hardening of polypropylene increases as the concentration of long chain branched PP augments [55]; strain hardening of polypropylene containing organomodified clays decreases as the clay content increases [54]; and strain hardening of high density polyethylenes enhances when the crosslinking density (provoked by electron beam irradiation) is increased [53].

## 3. Thermoset Polymers

Thermosets are very interesting materials from a rheological point of view, as the viscosity changes drastically from the initial, purely viscous formulation to the final material, which is a solid achieved by the curing process. From the point view of processing, the initial formulation should have a low enough viscosity to facilitate flow during moulding, but appropriate to avoid filler settling in the case of composites. Once the basic formulation has filled the mould, it is heated resulting in the chemical reaction that brings about a solid formed by a three-dimensional network, constituted by covalent bonds. Therefore, in the case of thermosets, the processing step and the achievement of the final material occur at the same time, as once the three-dimensional network is formed, the material can no longer be shaped. Thermosets represent only 10% of all the polymers produced, but they are essential in automotive and aerospace industry due to their outstanding mechanical properties and thermal and chemical resistance [56,57,58]. 

In order to obtain the thermoset solid material with the wanted shape and properties, three key parameters are involved: viscosity of the formulation, curing process conditions, and cross-linking density [56,59]. As mentioned, the initial viscosity has to be low to properly fill the mould, but also adequate to wet the fillers (or nanofillers) in the case of composites, or to be applied easily to surfaces in coatings. Regarding curing process, the goal is to have a short curing time with the lowest possible temperature to reduce energy consumption. The curing process is monitored in preference by rheological techniques, although other techniques, such as DSC, are also employed. The advantage of rheological measurements lies with their sensitivity to even very small molecular weight changes. During curing, the initially very low viscous formulation starts polymerizing, thus increasing its molecular weight, until a solid-like material is obtained, in which a three-dimensional network is constituted by covalent bonds between polymer chains [60,61]. The transition from the liquid-like material to the solid-like material is known as the gel point; at this point, an irreversible change occurs, as an insoluble material is formed [57]. Different strategies have been developed in the literature to determine the gel point [60]. Once a rubbery or glassy material is obtained, the cross-linking density can be calculated from the elastic modulus by employing the rubber elasticity theory [61]. Estimating this parameter is crucial, as it governs the final properties of the thermoset material [61].

In Spain, significant effort has been devoted to the understating of thermosets in the last decades. This is reflected in the publication of 84 papers dealing with rheology of thermosets in the last 25 years (Table 3).

The Rovira i Virgili University (Tarragona) accounts for almost 50% of the papers which match thermosets analysis with rheological techniques. The POLTEPO group of this university, led by Professor Angels Serra, is centred on the analysis of the processability, curing process, and cross-linking density of thermoset formulations. Outstanding research has been carried out by the group on the improvement of thermosets, employing new initiators [62,63] and developing dual curing procedures [64,65]. They have also studied the effect of adding polymers with different chains topology [66], as well as copolymers [67]. According to the Web of Science, the paper of Más et al. [67], of POLTEPO group, is the most cited among Spanish papers on the rheology of thermosets processing to date. Currently the group is implied in the development of advanced thermosets, including shape memory and conductive materials, as well as bio-based systems. 

**Figure 6:**
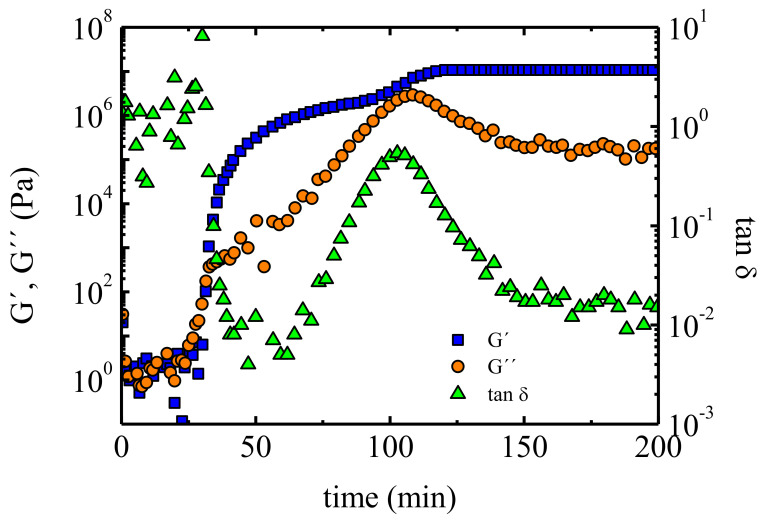
Storage modulus, loss modulus, and tanδ as a function of time for the formulation without star copolymer, DGEBA/methyl tetrahydrophthalic anhydride/benzyl dimethyl-amine [68] Reprinted with permission from [68] Copyright 2014 Elsevier B.V.

The effect of the different components of the formulation on the processability has been studied by Professor Serra and collaborators. For example, poly(glycidol)-b-poly(ε-caprolactone) linear and a multiarm star block copolymers have been added to diglycidylether of bisphenol (DGEBA), with the aim of improving the properties of the latter [68]. SAOS results reveal an increase in the complex viscosity of almost two orders of magnitude, with respect to pure DGEBA, when a 10% in weight of linear copolymer is added. Nevertheless, the addition of a multiarm star copolymer gives rise to only a slight increase in the viscosity and no worsening of the processability, whereas the mechanical properties are improved. Star copolymers have been also employed to modify the properties of diglycidylether of bisphenol A/methyl tetrahydrophthalic anhydride/benzyl dimethylamine formulations. The gel time has been determined using SAOS isothermal experiments. In Figure 6 G’, G” and tanδ are shown as a function of time for a sample cured at 100 °C and measured at a frequency of 5 Hz. There is a crossover between G’ and G” at 30 min, which marks the transition from a liquid-like material to a solid-like one and, therefore, this crossover can be used as a measure of the gel time. However, the G’ and G” crossover depends on frequency, so it is more appropriate to employ the tanδ crossover, which can be obtained by measuring tanδ versus time at different frequencies; if the curves obtained at different frequencies are plotted together, a crossover is observed at a certain time, i.e., at the gel time. All tanδ curves show the same value, independent of the frequency at which the measurement was performed. Acebo et al. [68] have used this method to determine the gel time of different samples. In addition, they have determined the vitrification point. Vitrification occurs when glass’ transition temperature approaches the reaction temperature, so at this point the reaction cannot proceed. Vitrification point is equivalent to the time at which G” and tanδ show a maximum, and in the case of G’ an increase is observed; for the material studied by Acebo et al., this occurs at 100 min. According to the obtained results it can be asserted that the addition of the star copolymers only subtly increases the gel time, not significantly altering the curing process.

The analysis of the crosslinking density by means of Dynamic Mechanical Thermal Analysis, DMTA, is also in the field of interest of the researchers of the POLTEPO group of the Rovira i Virgili University. For instance, taking advantage of the elastic modulus in the solid state obtained by DMTA, the rubber elasticity theory is used to calculate the crosslinking density of different formulations of DGEBA with different content of s(γ-butyrolactone) [69]. The calculated average molecular mass between crosslinks, M_c_, increases with butyrolactone: pure DGEBA has a *M_c_* of 338 g/mole face to 485 g/mole for the sample with the highest butyrolactone amount, 1:1.

**Figure 7:**
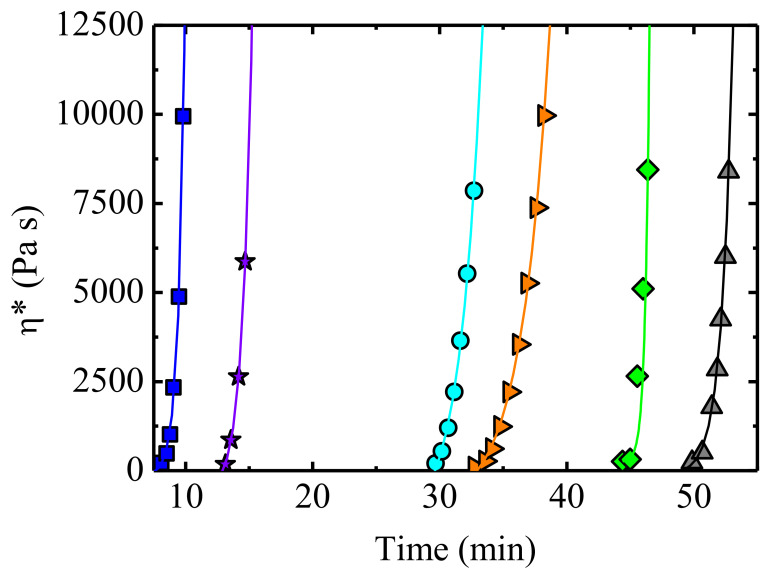
Complex viscosity as a function of time for different DGEBA/MXDA formulations. Square neat = DGEBA/MXDA, star = 5 wt % PSEO (block copolymer), circle = 5 wt % PSEO and 30 wt % HBC (liquid crystal), inverse triangle = 30 wt % HBC, diamond = 5 wt % PSEO and 50 wt % HBC, and triangle = 50 wt % HBC. Gelation times are obtained from the asymptotes (see text) [72] Reprinted with permission from [72] Copyright 2008 Acta Materialia Inc. Published by Elsevier Ltd.

At the University of the Basque Country, it is compulsory mentioning the work of Professor Iñaki Mondragon in the field of thermosets. Within his excellent career, Professor Mondragon, who passed away in 2012, made milestone contributions to polymer science and technology, and created and directed the Materials and Technology Group of this university. Within the framework of this review, we note his research on the improvement of epoxy resins adding different thermoplastics [70] or nanoparticles [71]. The effect of these components in the curing process was studied employing rheological techniques. Professor. Mondragon and his group also worked in the development of nanostructured thermosets, employing for that different block copolymers, liquid crystals and conductive nanofillers. We note the development of thermoresponsive meso and nanostructured thermosets employing DGEBA, and an aromatic amine hardener, m-xylylenediamine (MXDA) [72]. The addition of poly(styrene-b-ethylene oxide) block copolymer (PSEO) results in the microseparation of the liquid crystalline phase, which leads to a thermoresponsive material capable of switching from opaque to transparent state. The results of the curing process of these systems, monitored by SAOS tests, are shown in Figure 7. For the DGEBA/MXDA blend, a sudden increase in the viscosity is observed at 9 min; however, when a considerable amount of liquid crystal (30 or 50 wt %) is added, the upturn of the viscosity is shifted to longer times, reflecting a significant increase in the curing time. The gelation time is obtained from the asymptote of the viscosity.

Thermosets containing conductive nanoparticles have been studied by Professor Antxon Santamaria from the University of Basque Country in collaboration with TECNALIA Research and Innovation Center [73,74,75]. One of the pillars of this collaboration is the rheological analysis of nanocomposites elaborated dispersing electrically conductive carbon nanotubes, CNT, in thermosetting epoxy resin matrices. Viscosity before curing is measured to evaluate the CNT content needed to form a percolated network, and to study the processability of the nanocomposite [73]. In this context, the conditions to manufacture multiwall carbon nanotubes (MWCNT)/benzoxazine buckypapers have been studied by Chapartegui et al. [74]. The role played by rheology in buckypapers has been highlighted by these authors, allowing selecting the best time and temperature conditions to infiltrate the resin through the nanoparticles network. SAOS measurements were performed to study the curing process, concluding that the presence of MWCNT reduces the time needed to reach the gel point, although this effect is less significant for concentrations above the percolation threshold. The accomplished research, based at great extent on rheological results, leads to thermosets with outstanding electrical properties [74].

In the last years, the development of biobased thermosets has gained attention, triggered by the need for a sustainable progress. Recently, the group led by Professor Julio San Roman at the Spanish National Research Council (CSIC) has developed a fully biobased material employing maltodextrin and two different polycarboxylic acids: citric acid and tartaric acid [76]. Citric acid contains three carboxyl groups and one hydroxyl group, whereas tartaric acid contains two carboxyl groups and two hydroxyl groups; this affects the crosslinking of the sample and the hydrogen bond interactions. Applying a thermal treatment to the sample a polycondensation reaction occurs between the maltodextrine and the acid. SAOS measurements monitoring the modulus as a function of temperature indicate that the crosslinking temperature is higher for tartaric acid than for citric acid composition. On the other hand, the formulation with 80% of maltodextrin shows a significant storage modulus increase, which results from the hydrogen bonds interactions of maltodextrine molecules. Isothermal SAOS experiments of formulations containing different amounts of maltodextrin show that the gel time is shorter for the formulation containing citric acid. Therefore, using rheological methods, the higher reactivity and higher crosslinking capability of citric acid, as compared to tartaric acid, is demonstrated. 

## 4. Adhesives

The term *processing* typically associated to plastics is rarely employed in the field of adhesives. Notwithstanding, the application of adhesives to produce welding between different parts of any material assimilates a process with clear concomitances with what we understand as polymer processing. This is quite evident in the case of very popular hot-melt adhesives based on semi-crystalline polymers, whose requirements are: (a) Low viscosity for easy extrusion in the gun, as well as to wet the welding surface; (b) Good immediate adhesion (tack) at the application temperature; and (c) Solidification (crystallization) at room temperature to bring about permanent adhesion. 

In the case of pressure sensitive adhesives (PSAs) solidification at room temperature is not required, as the utility of this kind of adhesives lies in their persistent tack. On the other hand, for structural adhesives, in which permanent adhesion owes to an irreversible curing process (cross-linking), the application and use resembles the processing of thermosetting polymers. In this case, the curing time and cross-linking density are the most important features.

While the rheological implications of an adhesive when it flows in the gun and when it wets the welding surface are obvious, the viscoelastic foundations of immediate adhesion or tack are less conspicuous. A measure of tack is given by the energy dissipated in the debonding process in the so-called probe-tack tests. Based on the works of Creton [77] and Piau [78], the liaison between the results of tack and those of viscoelasticity can be summarized as follows: (a) A so-called “viscous adhesion” characterized by a force which goes rapidly to a maximum and then decreases slowly to zero, giving a low adhesion energy, and (b) A “viscoelastic adhesion” pattern, characterized by a force decrease to a plateau value, associated to a fibrillation process, with a higher adhesion energy which is measured as the area under the stress–strain debonding curve.

Under the above-mentioned premises, the number of papers which imply rheological results published by Spanish scientists in the last 25 years is estimated to be 63, distributed historically as depicted in Table 4.

The most relevant scientist in Spain in the field of adhesion and adhesives is Professor Jose Miguel Martín-Martínez from the University of Alicante (UA). He founded the Adhesion and Adhesives Group of UA in the nineties, and has collaborated closely with the Spanish Footwear Technology Centre (INESCOP) merging academia with industrial applications of adhesives. The role played by rheology in adhesives has been extensively studied by Martín-Martínez; he has published 43 of the 63 papers which are numbered in Table 4. The majority of his papers which include rheological aspects of adhesion refer to semi-crystalline polyurethanes used, indistinctly, as hot-melt, solvent based, and waterborne adhesives. 

The most elemental aspect of the rheology of adhesives, that is to say, viscosity results linked to application and wetting capacity, is extensively considered by Martín-Martínez. In particular, the relationship between the adhesive formulation and its viscosity is systematically treated by him. For instance, the alteration of the viscosity results of polyurethane adhesives by the addition of inorganic fillers is contemplated in many papers within his career. References [79,80,81,82] constitute examples separated in time. Additionally, the influence of the so-called tackifiers on the viscosity of waterborne and hot melt polyurethane adhesives [83,84,85] and acrylate based adhesives [86] has deserved his attention. On the other hand, the effect of phase separation on viscosity is reported, for instance, in References [87,88] where different hard segments contents are considered varying the diisocyanate/macroglycol (NCO/OH) molar ratio, observing a lower viscosity as the NCO/OH ratio is decreased. In another paper, it is reported that the ionic group content in polyurethane ionomer structure controls the hard segment proportion, which affects the resistance to flow under temperature [89]. 

Frequency sweeps in small amplitude oscillatory shear (SAOS) experiments, often simply called dynamic viscoelastic measurements, are profusely employed to investigate adhesives processing and applications. The aim of these kinds of experiments is to establish relationships between the values of the elastic and viscous moduli at different frequencies and temperatures, and adhesive performance in terms of tack and peel testing in the solid state. At the University of Alicante and INESCOP, dynamic viscoelastic measurements in the solid state (Dynamical Mechanical Thermal Analysis, DMTA) as well as in the liquid state have been performed, but in this review paper we only refer to the latter, as DMTA is rather used for basic characterization, such as phase separation in polyurethanes. 

**Figure 8:**
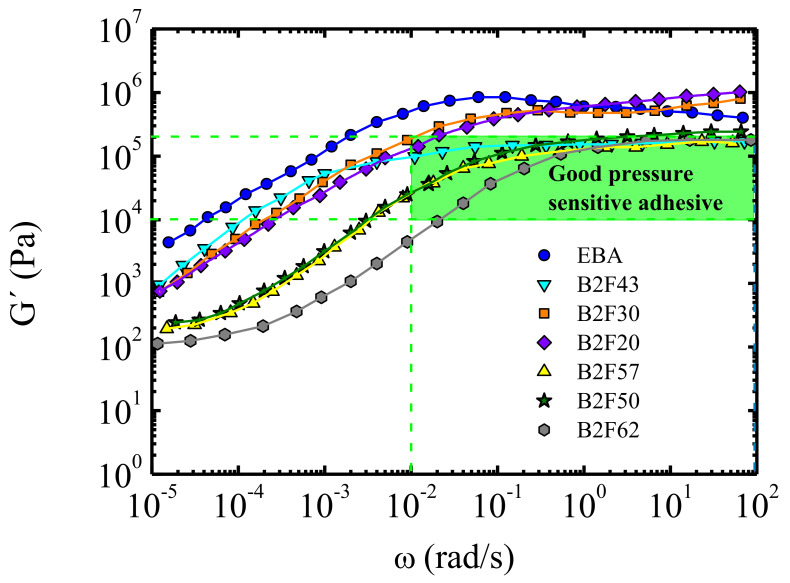
Storage modulus versus frequency for ethylene-co-n-butyl acrylate-based formulations; the number next to F indicates the amount of hydrogenated glycerol rosin ester in weight. As indicated, the square marks the quality of the pressure sensitive adhesive [91].

Among different papers published by both institutions on this subject [79,81,90,91,92,93,94,95,96,97,98,99] we remark the paper of Sancho-Querol et al. [91]. Figure 8, taken from this paper, is representative of the usefulness of dynamic viscoelastic results, in particular elastic (storage) shear modulus, G’, versus frequency, to ascertain the quality of ethylene-co-n-butyl acrylate-based formulations as pressure sensitive adhesives. The Dahlquist criterion [100] is used in this figure to establish good performing pressure sensitive adhesives formulations. This criterion proposes that the tensile compliance *D* (*t*) should be of the order of 10^−6^ Pa^−1^ or larger on the time scale of the bonding step (typically 1 s), which is equivalent to *G’* < 3.3 × 10^5^ Pa at a frequency of 1 rad/s, to obtain good immediate adhesion or tack. Interestingly, in this reference, the lesser-known Chang [100] approach is also used. Accordingly, viscoelastic windows are obtained by plotting the values of G’ versus G” at frequencies of 0.01 rad/s and 100 rad/s, which are associated with the bonding and debonding processes of the pressure sensitive adhesives, respectively [91].

In addition to rheological analysis of hot melts, waterborne polyurethanes, and pressure sensitive adhesives, rheological measurements applied to other kinds of adhesives have been investigated by Martín-Martínez and his group. For instance, a series of acrylic adhesive mixtures formulated for use in strabismus surgery were studied by means of SAOS tests [101].

Other groups in Spain have also investigated on the rheology of adhesives, but much less extensively than the Adhesion and Adhesives Group of the University of Alicante. The research, led by Professor Antxon Santamaria at the University of the Basque Country (UPV/EHU), is centred on the viscosity and SAOS results of polyurethane and carbon nanotubes (CNT) composites, apt as hot melt adhesives [102,103,104]. The main objective of these papers is to study the effect of adding carbon nanotubes to polyurethane, on the rheology and adhesion of electrically conductive hot melt adhesives. It is demonstrated that, although the terminal viscoelastic zone is severely affected by the presence of CNTs, the viscosity at the shear rates involved in the extrusion flow at the gun is not significantly increased, making the application of the nanocomposite adhesive easy. The analysis of the SAOS results indicate that the alteration of the values of the elastic and viscous moduli at low frequencies (with G’ > G”) does not affect tack results, as the aforementioned Dahlquist criterion is fulfilled. The liaison between SAOS results and tack results is confirmed in two more papers of this group, on polyurethane solutions [105] and styrene-butadiene-styrene (SBS) block copolymers [18], respectively. In both papers it is stated that tack only occurs in formulations which have an elastic shear modulus below 3.3 × 10^5^ Pa (Dahlquist criterion).

Relevant papers which contain rheological results of bio-sourced adhesives have been published by the group of Professor Jose María Franco from the University of Huelva. In one of the works [106], cellulose acetate (CA) is modified with diphenylmethane-4,4’-diisocyanate (MDI) at different NCO:OH molar ratios, and the resulting biopolymers are mixed with castor oil (CO) at 1:1 wt ratio. It is reported that the best thermo-rheological behaviour (as studied by G’ and G’’ plots at different frequencies and temperatures), and adhesion on wood and stainless steel is obtained for the formulations which show the higher compatibility between the hard and the soft microdomains. Similar thermo-reological analysis is made in another paper from the group [107].

Dynamic viscoelastic results in the liquid state are also contemplated in a paper [108] where solvent free adhesives derived from castor oil are analysed, observing a correlation between the elastic shear modulus and an adhesion energy obtained through tack experiments. A comparison of the performance of bio-sourced polyurethane adhesives with commercial adhesives is carried out [109], analysing SAOS results of the different samples. 

The rheological implications of structural adhesives, which should cure or crosslink to give a permanent and strong welding, have been rarely considered in the research activity in Spain. Only studies carried out, by Dr. Carlos Gracia from TA Instruments in Madrid and researchers in TECNALIA Research and Innovation Center in the Basque Country, about the monitoring of the curing process by dynamic viscoelastic measurements, have been published in scientific journals. In a couple of papers [110,111], plate–plate oscillatory rheometry results are used to compare the gel time and activation energy in the curing process of different epoxy systems. Additionally, rheological analysis and dielectric analysis are compared [111]. On the other hand, a viscoelastic analysis of the curing process of an adhesive based on tetrahydrofurfuryl methacrylate and ethoxylated aromatic amine and benzoyl peroxide, as compared to commercial adhesives triggered by water, is reported by authors of the group led by Dr. Gracia [112]. In a pioneering work from the same group [113], the heat generated by the Joule effect in a (MWCNT)/epoxy electrically conductive nanocomposite triggers the curing process, which is monitored by rheological tests. 

In a work carried out in TECNALIA [114], three different kinds of inorganic nanoparticles are incorporated into a thermoset polyurethane adhesive, observing, among other features, their effect on curing time. The gel time is taken as the point at which the elastic modulus, G’, crossovers the viscous modulus G’’. It is observed that in all cases the gelation process is fastened when nanoparticles are incorporated to polyurethane.

## 5. Biopolymers

The growing concern about the environment in recent years has resulted in the development of biobased and biodegradable polymers which target two of the main problems of plastic industry: the dependence of fossil fuels and the high volume of generated waste. Biobased polymers are defined as polymers which can be obtained from renewable sources. Biodegradable polymers refer to polymers that can be degraded under some specific conditions resulting in CO_2_, water, and biomass, among others. In the literature, a great effort has been made to substitute common polymers with materials derived from sustainable sources. Biopolymers with applications in a wide range of fields have been developed, such as adhesives, lubricants, packaging, and plastic parts in general [115,116,117]. Depending on the application, different rheological features are required. 

In Spain, several researchers have studied the rheological and processing properties of biopolymers. In total, 50 papers have been published in the last 25 years; 42 of them deal with thermoplastics, 6 with biocomposites, and 2 with bioadhesives. A total of 72% of the papers werepublished in the period 2015–2020. The study of rheology-processing relationship focused on biopolymers has been mostly led by the Universities of Sevilla (Professor Antonio Guerrero) and Huelva (Professor Críspulo Gallegos). Both universities account for 43 papers of the 50 published on this topic in Spain.

The ReoTech group, directed by Prof. Antonio Guerrero, has deeply studied biopolymers based on proteins and polysaccharides [118,119,120,121,122]. In particular, rheology and processing of hydrogels [123] and processing of biopolymers with potential applications in packaging [124], superabsorbent materials [125], and scaffolds [123], has been considered. The paper of Bengoechea et al. [119] is currently the most cited in the field of the rheology of bioplastics in Spain, according to the Web of Science.

Guerrero’s group has developed an albumen/soy biobased plastic, which is constituted by proteins. Proteins are three-dimensional structures which are stabilized and strengthened by intermolecular forces, such as hydrogen bonds or hydrophobic interactions, among others. This kind of material can be processed employing conventional thermoplastic processing methods. To reach an adequate processing behaviour, proteins are mixed with plasticizers to reduce the intermolecular forces, increase the mobility of the chains, and obtain a dough-like material [122,126,127]. Extrusion or compression moulding techniques have been used, although one of the most relevant techniques to obtain plastic parts, i.e., injection moulding, has not been studied in detail, to date. Felix et al. [128] have focused on this issue analysing an albumen/soy biobased plastic.

**Figure 9:**
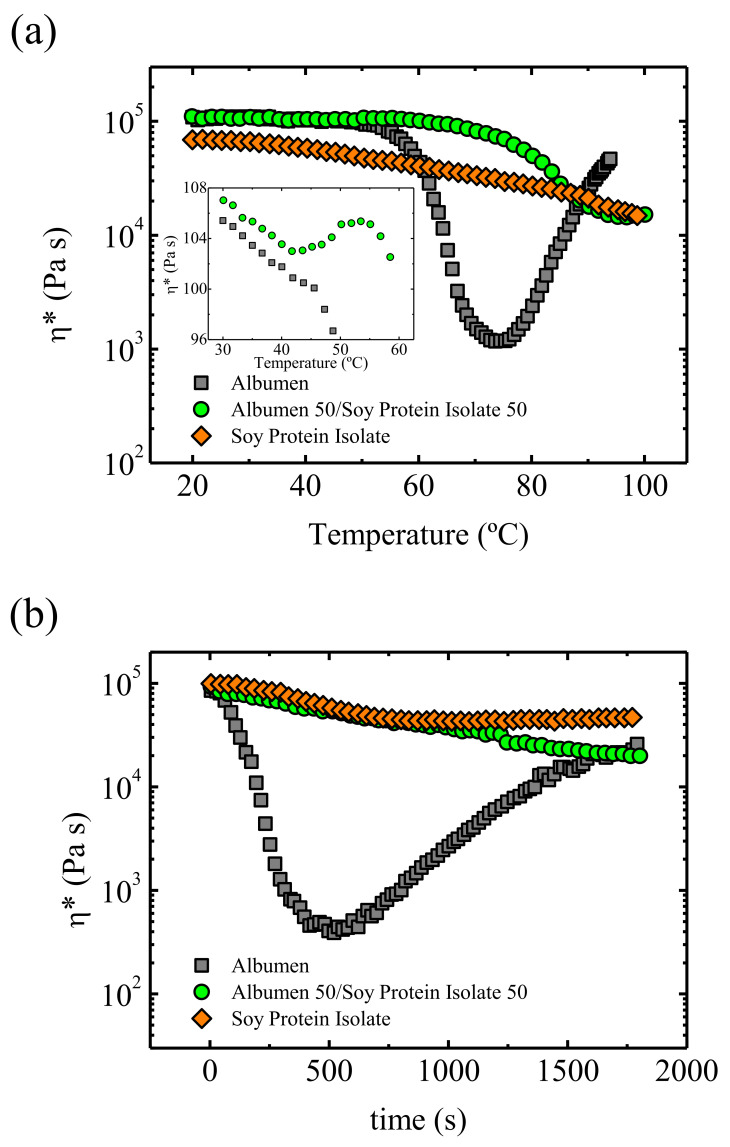
Complex viscosity of biobased albumin, soy protein, and albumin/soy blend as a function of (**a**) temperature and (**b**) time, to select suitable injection conditions (see text) [128] Reprinted with permission from [128] Copyright 2013 Elsevier Ltd.

Usually, biopolymers show a brittle behaviour, so in order to increase ductility chains, mobility should be increased using adequate plasticizers. For this purpose, Felix et al. [128] employed glycerol, preparing protein/glycerol blends in a 40/60 proportion. The DSC results reveal the presence of two relevant transition temperatures: the glass transition temperature, *T_g_*, and the denaturation temperature at which protein aggregation and cross-linking occurs. Small amplitude oscillatory shear, SAOS, experiments have been performed to select the most appropriate processing conditions. First, viscosity as a function of temperature is measured, to select a temperature at which the viscosity is appropriate for injection moulding. In the case of albumin/glycerol blends the viscosity shows a minimum around 75 °C, followed by an increase due to denaturation of proteins. In addition, isothermal SAOS experiments have been conducted, analysing the effect of time to establish the appropriate residence time avoiding denaturalization of the proteins. Albumin/glycerol system shows an inflection point at 100 s, which is the time needed to reach the *T_g_* of the system, and a viscosity minimum at about 500 s. Then, an increase in the viscosity is observed, which corresponds to the denaturation of the protein. Considering the evolution of the complex viscosity as a function of temperature and time (Figure 9), Felix et al. [128] were able to select the adequate temperature to inject the material in the mould. Good, injected bioplastic parts can be obtained, although their mechanical properties are poorer than those of LDPE standards.

Jerez et al. [120,122] developed gluten-based biomaterials, studying thoroughly the effect on rheological properties of different factors, such as drying time for the sample prepared by casting, plasticizer (glycerol) content, mixing speed, and thermal history. A reduction in the elastic and viscous moduli is observed as glycerol content is increased, as the intermolecular interactions between protein chains are lessened. Measuring the torque as a function of time, Jerez et al. have studied the materials at different stages of processing, observing an increase in the elastic modulus of one order of magnitude, which reflects the development of a gel-like behaviour. The elastic modulus is higher for the sample processed mechanically in comparison to that obtained by casting, due to the high temperatures reached during processing which result in denaturation and crosslinking.

Another issue that has attracted the interest of Spanish researchers is the development of biobased lubricant greases. Greases are viscoelastic materials; depending on temperature and applied stress, among other factors, they can show from solid behaviour up to liquid-like behaviour, if the yield stress is surpassed [129,130,131]. Usually, greases are employed as wheel bearing lubricants, substituting oil when high temperatures are required. Greases are colloidal suspensions constituted by oil (synthetic or mineral), which is the main component of the formulation, and a thickener that forms a three-dimensional network to trap the oil. The grease can also contain other additives to improve its performance. 

Professors Críspulo Gallegos, José María Franco, and Concepción Valencia from the University of Huelva have developed biobased greases [132,133,134,135]. As an example, a completely biobased grease has been obtained employing castor oil (65 wt.%) and acylated chitosan (35 wt.%). Small amplitude oscillatory shear experiments reveal that for this grease the elastic modulus, G’, governs over viscous modulus, G”, giving rise to a plateau at intermediate frequencies. The same viscoelastic behaviour is observed for the greases based on calcium and lithium. The plateau modulus value is defined as the value of the elastic modulus at the frequency at which tanδ shows a minimum. It is observed that the plateau modulus of the biobased grease is more susceptible to the effect of temperature. 

Continuous flow experiments show that both types of greases have similar viscosity values, although the biobased grease shows a higher dependence on shear rate. The destruction and recovery of the structure has been investigated, applying different shear stress programs. The results indicate that the biobased grease shows a very low destruction degree, whereas the recovery is higher for the lithium-based grease. 

Interest has also been centred on biobased thermoplastic polymers, as is reflected in several papers dealing with rheological and processing properties of polylactic acid (PLA) and some biobased blends [36,136]. The work lead by Professor Maria Lluisa Maspoch, from Centre Català del Plàstic (CCP) and Polytechnic University of Catalonia (UPC), is focused on the processing of PLA [136,137], with allusions to rheological features. This is the case of the study carried out on the evolution of the torque in an internal mixer during the reactive processing of PLA: The observed increase in the torque is linked to crosslinking and branching reactions. In particular, using SAOS experiments, Cailloux et al. [136] have monitored the complex viscosity during time in a reactive extrusion calendaring process. The addition of a reactive agent results in an increase in the complex viscosity, reaching a constant value after 20 min when an internal mixer is employed; this constant value reflects the saturation of the reactive species. For the extrusion calendaring mixing process, a longer time is needed to reach stabilization, as in the extrusion there is a continuous discharge of the material.

Taking advance of SAOS measurements, Cailloux et al. have investigated other rheological features of PLA samples processed by internal mixing and by extrusion calendaring process [136]. The temperature–frequency master curves of the complex viscosity for samples containing a reactive agent at a reference temperature of 180 °C show several curvatures, which are associated to different relaxation times due to the presence of branched chains. The results indicate that the samples processed by extrusion and calendaring show longer relaxation times than those processed in the internal mixer. To confirm the effect of branching on the relaxation times, Cailloux et al. have also considered Mavridis–Shroff–Van GurpPalmen plots [138,139,140], as well as creep experiments to obtain the relaxation time spectra. It is observed that neat PLA shows a single relaxation mechanism, whereas the samples with the reactive agent show several peaks and longer relaxation times compatible with the presence of branches.

## 6. Polymer Composites and Nanocomposites

The development of polymer composites and nanocomposites has attracted the interest of many researchers, as the addition of fillers and nanofillers can improve the mechanical, barrier, and thermal properties of polymers in general. Particularly interesting is the case of conductive nanofillers which, properly mixed with a polymer matrix, bring about semiconductor materials, opening new routes in polymers research. Nevertheless, the presence of fillers can hinder the material processing, due to an observed viscosity increase. Therefore, evaluating the effect of fillers on the rheological properties from a processing point of view is of paramount importance. However, as rheology is a valuable tool to characterize the dispersion of the particles in a polymer matrix [141,142,143,144], most of the literature is rather focused on the relationship between the rheological properties and the structure of the composites/nanocomposites. When a filler is added to a polymer matrix, a transition from a liquid-like behaviour to a solid behaviour is observed, above a certain concentration. This concentration is known as the rheological percolation threshold, and it is assumed to stand for the constitution of a polymer-filler network. Small amplitude oscillatory shear, SAOS, measurements are employed to determine this percolation threshold. However, from the processing point of view, it is more relevant to study the viscosity as a function of shear rate in continuous flow, to analyse the viscosity enhancement with respect to neat polymer caused by fillers. For instance, the formation of a percolated network results typically in a viscoplastic behaviour, i.e., a progressive increase in the viscosity as the shear rate tends to zero [141,142,143,144] which can affect certain processing methods, such as compression moulding. More detailed studies can be carried out in extrusion flow, also analysing the extrudate swell at the exit of the die, or the effect of pressure on viscosity which is pertinent considering that, during extrusion and injection moulding, the polymers can be submitted to high pressures.

In Spain, the rheology of composites and nanocomposites has drawn considerable attention, notwithstanding the majority of the works refer to the analysis of the relationship between rheology and micro/nano structure, also evaluating the rheological percolation threshold. The works focusing on rheology related to processing are more scarce: according to our records, 63 papers have been published in the last 25 years, as is depicted in Table 5. The distribution of these papers correlative to the polymer material is: 27 on thermoplastics, 20 on thermosets, 9 on adhesives, 4 on bitumens, and 3 on biopolymers. 

The research of Professor Santamaria from the University of the Basque Country is centred on the relation *structure-rheology-processing* in nanocomposites [145,146,147] and polymer blend nanocomposites (PBNANOs), analysing the information extracted from small amplitude and large amplitude oscillatory shear (SAOS and LAOS) measurements [148,149,150], but also contemplating extrusion flow experiments. 

Santamaria’s group has studied the rheological properties of Polyethylene terephthalate/Low Density Polyethylene/Titanium dioxide (PET/LDPE/TiO_2_) PBNANOs [151]. The morphology of these ternary systems is characterized by LDPE droplets dispersed in a PET matrix and the titanium dioxide particles located at the interface. The study of the viscosity as a function of shear rate shows that, in this case, the presence of titanium dioxide reduces the viscosity of the system at intermediate and high shear rates, acting as a processing aid agent. However, analysing the low shear rate range, an upturn of the viscosity is observed (viscoplasticity) for some of the blends containing TiO_2_. This aspect is relevant for some processing techniques such as thermoforming, in which viscoplasticity can avoid sagging of the polymer sheet. An analysis of the factors that determine viscoplastic behaviour in these PBNANOs is presented.

The issue of the dependence of the viscosity on pressure has been addressed in a couple of papers published by the aforementioned group of the University of the Basque Country [9,152]. As an example, the study carried out with poly(styrene) (PS), PS/multiwalled carbon nanotubes (MWCNT) and PS/graphene is remarked. Rodriguez et al. [30] have calculated the pressure coefficients for Newtonian and non-Newtonian viscosities, observing that they are similar for the PS matrix and the nanocomposites at the shear rates involved in extrusion and injection moulding. The piezorheological simplicity/complexity has been also studied, concluding that PS/MWCNT is piezorheologically simple at intermediate and high shear rates, in which the material shows a shear thinning behaviour. On the other hand, PS/Graphene is piezorheologycally complex in the entire shear rate range, that is to say, in the linear and non-linear zone. According to the authors this complexity can arise from the ability of polymer chains to anchor to the surface of the graphene platelets. 

A sound study of nanocomposites has been led by Professor María José Abad at the Coruña University. The research is focused on the relationship between rheology and structure of nanocomposites [153,154,155] and PBNANOs [156,157], but the viscosity of these systems as a function of shear rate is also analysed under the perspective of processing. We remark that the paper of Pardo et al. [154] of this university is the most cited (Web of Science) among the Spanish papers on the rheology of polymer composites processing.

Among others, the group has studied an interesting composite constituted by electrically conductive polyanyline (PANI) and ethylene-vinyl acetate (EVA) [156], in which PANI is in the solid state due to the low temperatures employed. The electrical conductivity of some of the blends is of the order of pure PANI. In fact, PANI acts as a filler, increasing the viscosity. In order to improve the compatibility of the systems, gallic acid is used as a compatibilizer, observing an increase in the viscosity which results from the better dispersion of PANI and the improved adhesion between PANI and EVA. According to the paper of Dopico-García et al. [156], hydrogen bonds are formed between EVA and the compatibilizer enhancing the adhesion between PANI and EVA phase.

**Figure 10:**
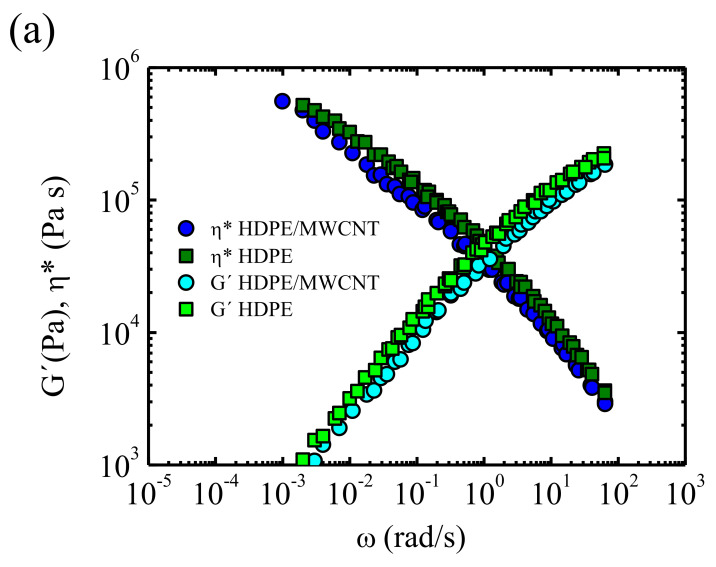

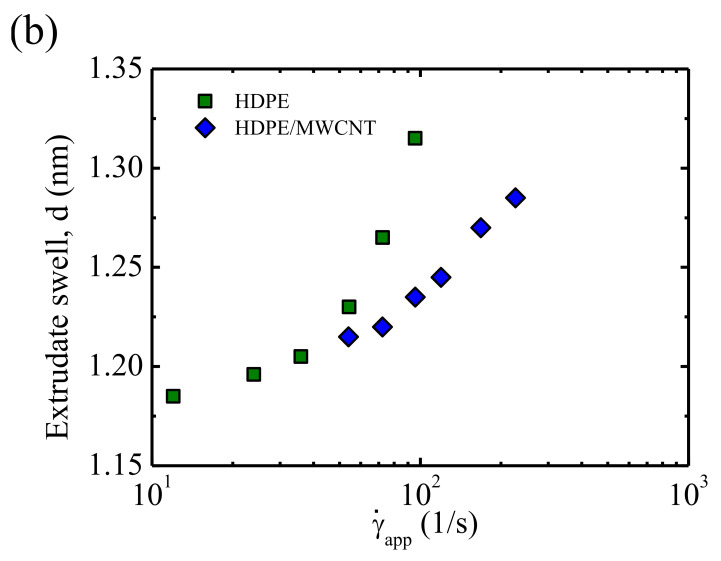
(**a**) Storage modulus and complex viscosity as a function of frequency for neat HDPE and HDPE/MWCNT. (**b**) Extrudate swell as a function of apparent shear rate for neat HDPE and HDPE/MWCNT nanocomposite [158] Reprinted with permission from [158] Copyright 2009 American Chemical Society.

A thorough study focusing on the processing and rheology of high-density polyethylene HDPE/MWCNT has been carried out by Juan Francisco Vega and coworkers at the Spanish National Research Council (CSIC). Vega et al. [158] have studied nanocomposites constituted by bimodal HDPE with low MWCNT content (0.52 wt %). The measurements carried out in the linear viscoelastic region indicate that a percolated network is not formed, as a liquid-like behaviour is observed at low frequencies (absence of an elastic modulus plateau). Thus, the MWCNT concentration employed in the work is below the rheological percolation threshold. In addition, the nanocomposites exhibit a lower complex viscosity and storage modulus than neat HDPE (see Figure 10). This owes to the adsorption of HDPE chains with longest relaxation times onto the surface of CNTs, which results in a lower apparent molecular weight of the polymer matrix and, thus, in reduced entanglement density. The viscosity as a function of shear rate was also investigated observing a good overlap between the results obtained by oscillatory shear experiments and capillary flow (Cox Merz rule). Usually, the Cox Merz rule is not fulfilled for nanocomposites, as a three-dimensional network that breaks at high shear rates is formed. However, in this case the concentration of CNTs is below the percolation threshold which explains the obtained results.

The measurements performed in extrusion capillary flow by Vega et al. reveal that the so-called slip stick distortion regime is shifted to higher shear rates and stresses as compared to neat HDPE. Moreover, the analysis of the extrudates reveals that nanocomposites have less extrudate swelling (Figure 10), so better dimensional stability than HDPE. In summary, it can be asserted that, in this case, the presence of MWCNTs improves the extrusion capacity of the material, as it reduces the viscosity and amends surface stability. 

Fernández and co-workers [39] at the University of Zaragoza have developed a methodology to measure the rheological properties during injection process, as mentioned in the section of thermoplastics (Section 2.1). They have considered the plastification process of injection of recycled plastics that show unconventional characteristics, measuring the pressure and temperature at the end of the barrel. According to Hay and Mackay [159] the viscosities measured in a capillary or slit die rheometer do not match with the values measured in a torsional rheometer, as the flow is affected by slip, dependence of the viscosity on pressure, viscous heating, heat transfer, and entrance and exit effects. Therefore, it is important to be able to precisely determine the viscosity of the materials from the processing point of view. Interestingly enough, Fernandez and co-workers [39] have tested their methodology to measure the rheological properties during the injection process (described succinctly in Thermoplastics section) with a commercially available 12% talc filled PP (Hostacom CR 1171 G1A). The results show that mineral filled PP exhibits lower viscosity values when it is recycled.

## 7. Asphalt Binders and Polymer Modified Bitumens

Bitumen is a high boiling point viscous residue, mainly obtained from crude oils vacuum distillation, which is widely used as a binder of mineral aggregates in pavements. However, bitumen may find other multiple applications as waterproofing materials, joint sealant and for roofing membranes [160,161]. Its complex chemical composition consists of compounds with wide molecular weight distribution that can be broadly classified as maltenes or asphaltenes. Asphaltenes constitute the highest molecular weight and most polar fraction of bitumen. Viscoelastic properties of bitumen and, consequently, its performance in pavements strongly depended on the ratio of asphaltenes to the other constituents [162]. 

Bitumen properties are highly dependent on temperature, allowing its numerous applications. Nevertheless, this fact also represents its major weakness. Virgin and recycled polymers (e.g., waste crumb rubber) have been widely used to reduce thermal susceptibility and enhance bitumen performance [161,163,164,165]. At high in-service temperature, polymer modification leads to binders with enough stiff to withstand traffic loads, avoiding rutting or permanent deformation. Likewise, at low pavement temperatures, polymers endow bitumen with flexible characteristics able to avoid excessive thermal stresses and to endure the thermal cycling of service without cracking or deforming [161]. 

Accordingly, the industry has graded asphalt binders (neat and polymer modified bitumens) to ensure their applicability as a function of the environmental conditions, traffic density, etc. To that end, traditional specifications have characterized the consistency of bituminous binders by different standardized test (e.g., viscosity, penetration, ductility, and softening point). As a result, bitumen is selected according to its consistency at high temperature that, at the same time, ensures an adequate performance at low temperatures. However, a novel approach, stated in the USA since 1987 by the Strategic Highway Research Program (SHRP), evidenced rheology as a powerful and reliable technique for assessing mechanical properties of bitumen, which are directly related to binders’ in-service performance [166]. As a consequence, a set of performance-related specifications were developed, which were based on different rheological tests conducted on binders submitted to standardized aging conditions. Such specifications involve rotational viscometry (to measure bitumen Newtonian viscosity at high temperatures), small-amplitude oscillatory shear tests (to characterize bitumen linear viscoelastic properties at intermediate temperatures), and flexural creep (or uniaxial tension) tests in the linear viscoelastic region at low temperatures. Soon, this rheological approach was adopted in Europe.

In this context, the first article published by a Spanish group was entitled “Rheological characterisation of synthetic binders and unmodified bitumens” [167]. This research work dates back to 1999, and was performed in The University of Huelva within the scope of a research project studying the influence of composition and processing conditions on the bitumen properties modified with thermoplastic elastomers such as SBS, which also undertook the design of pigmentable synthetic bitumens with suitable rheological characteristics; binders with similar characteristics to bitumen but, for instance, with a greater easiness for its pigmentation. This paper is the most cited article within the scope of such non-bituminous binders, a topic that groups different articles published by Spanish researchers, and addresses the design of novel asphalt binders and roofing materials. 

In addition to the development of non-bituminous binders, other topics have focused on the interest of the Spanish publications involving rheology as a main tool for material characterization and product design. Among them, reactive modification of bitumens and the use of waste polymers have gathered most of articles published by Spanish research groups with, respectively, 33 and 37% of the total publications in relevant journals, according to the Web of Science. Modification by virgin polymers (involving different polyolefins or copolymers) and by the addition of fillers represent other categories with, respectively, 15 and 12% of the articles published. Finally, around 8% of the articles published by Spanish researchers address binder performance and aging assessment by means of rheological tools. 

As a whole, rheology applied to pavements and roofing materials has given rise to a fruitful production of articles, with more than 100 papers published in relevant journals (according to the Web of Science platform). Most of them have been arisen from three research groups, belonging to the Universities of Huelva, Basque Country, and Granada (Table 6).

Regarding the reactive modification of bitumen, most articles published address the excellent role of prepolymers functionalized with isocyanate groups (e.g., MDI-PPG and MDI-PEG type) or epoxy-derived polymers in the development of novel high-performance bituminous materials. As an alternative to crude oil-based polyols, such prepolymers may be also formulated from bio-based vegetable oils. Moreover, prepolymers functionalized with isocyanates have been used to be, simultaneously, bitumen modifiers and foaming promoters. As a result, resultant bituminous product may be designed to form either stable foams for isolation applications or unstable bituminous foams with the aim of reducing working temperatures in paving technologies. In addition, other chemical modifications by means of polyphosphoric acid, thiourea, or dodecenyl succinic anhydride, among other reagents, have been studied as adhesion promoters, rejuvenators and/or rheology modifiers. Related to this topic, the most cited articles were published by Martin-Alfonso et al. [168] and Navarro et al. [169,170], dealing with the use of isocyanate-functionalized prepolymers for paving and roofing materials. 

However, bitumen modification by polymeric wastes is probably the most relevant issue studied by Spanish researchers in terms of the total amount of citations received by the articles included in this topic (more than 1200 all around the word). This issue gathers numerous works involving modification with different reclaimed polymers (e.g., EVA, EVA/LDPE blends, PP, crumb tyre rubber (CTR), etc.). Most popular articles address the use of CTR and polyolefins as modifiers of bitumen thermo-rheological behaviour, and how their combined addition may lead to improved binder performance. In such mixtures, CTR mainly improves material behaviour at low in-service temperatures, whereas polyolefins mainly enhance high in-service temperature behaviour. Furthermore, mixing conditions, which should involve high shear and temperature, strongly affects the rheological behaviour and microstructure of the blend obtained, particularly in the case of polyolefins. Conversely, the processing device (i.e., shear conditions) has no influence on rheology and microstructure of CTR modified bitumens at temperatures below 180 °C, as break-up of the rubber crosslinked network was not observed under such conditions. In addition to the rheological behaviour, CTR digestion induced by processing strongly affects hot storage stability of the final binders, which is a major challenge for the development of these products. In this topic, most cited articles were published by Navarro et al. [165] and Garcia-Morales et al. [164,171], addressing rheology, microstructure, and storage stability of bitumens modified by recycled EVA, EVA/LDPE blends or CTR.

In addition to waste polymers, studies on rheological modification of bitumen by virgin polymers such as SBS, EPDM, HDPE, PP, m-LDPE, or EVA, among others, can be found published in relevant journals since 2002. Again, the effect of processing conditions, formulation, and destabilization mechanisms of polyolefins during binder hot storage have been widely studied. Regarding stability, improved stability was found by Gonzalez et al. [172] with metallocene catalysed LLDPEs (m-LLDPEs), if compared to conventional polyethylenes. Similarly, rheological properties of bitumen blends with metallocene catalysed atactic polypropylenes were studied for roofing materials. This study suggested polypropylene-saturated resin mixture may become the continuous phase, observing that the glass transition temperature of polypropylene decreased, and the pure bitumen broad relaxation disappeared. Such results were not obtained for atactic polypropylene [173]. Likewise, different works have studied bitumen modification as a function of EVA melting point, its vinyl acetate content (VA), and melt flow index (MFI). In this regards, low VA content was found to improve bitumen performance at medium-high in-service temperatures. On the other hand, binder viscosity at 135 °C decreased with the increasing MFI (no matter the selected VA content), which would facilitate polymer-bitumen mixing, mineral coating, and asphalt mix laydown/compaction at lower temperatures [174]. Finally, most cited articles in this topic dealt with rheology and stability of bitumen/EVA blends [175] and the effect of processing conditions [176]. 

On the other hand, the effect of inorganic filler concentration, composition, and size distribution on the rheology of bituminous mastics has been studied, where continuous phase may be either a neat bitumen or a polymer-modified binder. In this respect, Lagos-Varas et al. [177] found that the filler increases the rigidity of the mastic at all temperatures and frequencies tested, but the observed increase depends on the type of bitumen used. For LDPE-modified bituminous mastics, Roman et al. [178] found enhanced complex shear modulus and elasticity at high in-service temperature. Likewise, SBS-based mastic at 25 °C displayed an enhanced recovery back to linear complex modulus values, after being subjected to deformations in the non-linear viscoelastic region. Furthermore, novel approaches have studied bituminous mastics for pavements with improved fire performance [179], or mechanomutable asphalt binders able to modify their mechanical behaviour by means of a magnetic field, which were tested under cyclic creep and recovery loads [180]. Within this topic, a novel work which addresses mechanical and thermal properties of graphene modified asphalt binders has been one of the most cited articles [181].

A last group of papers gathers those articles assessing in-service performance, aging, or self-healing of bituminous binders and mastics by means of rheological tests. For instance, the effect of ageing and temperature on the fatigue behaviour of bitumens has been studied by Miro et al. [182]. Likewise, rheological properties of rejuvenated bitumens were studied by Romera et al. [183], which has been one of most cited articles in this category. 

## 8. Conclusions

The contribution to the rheology in polymer processing of Spanish scientists in the last 25 years (1995–2020) is highlighted, concluding that the interest on the subject is well extended. Papers published by 12 universities, 6 research institutes, and three private companies located in 9 of the 17 autonomous communities of Spain have been recorded.

Beyond the carried out numerical analysis of the published papers, a selection of the most relevant results, based on the rheological point of view of the authors, is presented.

## Figures and Tables

**Table 1:** Processing method and corresponding shear rate intervals.

Method	Shear Rates Range (s^−1^)
Compression moulding	1–10
Calendaring	10–100
Extrusion moulding	100–1000
Additive Manufacturing	10–2000
Injection moulding	1000–100,000

**Table 2:** Distribution of the papers published about rheology of thermoplastics processing in the period 1995–2020.

Institution	1995–1999	2000–2004	2005–2009	2010–2014	2015–2020
University of the Basque Country	8	8	9	4	10
Institute for the Structure of Matter CSIC (Madrid)	1	4	5	3	3
REPSOL and ERCROS	4	6	2	2	4
Others	1	1	3	5	12
In Total: 95	14	19	19	14	29

**Table 3:** Distribution of the papers published about rheology and thermosets in the period 1995–2020.

Institution	1995–1999	2000–2004	2005–2009	2010–2014	2015–2020
Rovira i Virgili University			8	17	14
University of the Basque Country	2	2	7	5	
Complutense University of Madrid				4	
National Institute of Aerospace Technology				3	
Others		1	5	7	9
In total: 84	2	3	20	36	23

**Table 4:** Distribution of the papers published about adhesives in the period 1995–2020.

Institution	1995–1999	2000–2004	2005–2009	2010–2014	2015–2020
Prof. Martin-Martínez	5	16	7	6	9
INESCOP			1	2	1
University of the Basque Country			1	3	1
University of Huelva				1	4
TECNALIA		1			
TA Instruments Spain			2		3
In total: 63	5	17	11	12	18

**Table 5:** Distribution of the papers published about rheology of the processing of composites and nanocomposites during 1995–2020.

Institution	1995–1999	2000–2004	2005–2009	2010–2014	2015–2020
University of the Basque Country		1		5	3
University of Alicante			1	3	1
Institute of Polymer Science and Technology, CSIC		1	1	4	
Coruña University				2	
Materials Technology Institute, Polytechnic University of València				2	
University of Zaragoza, Centro Politécnico Superior				2	1
University of Seville					3
Others		2	8	9	14
In total: 63		4	10	27	22

**Table 6:** Distribution of the papers published involving the rheology of bituminous binders in the period 1995–2020 in Spain (source, Web of Science).

Institution	1995–1999	2000–2004	2005–2009	2010–2014	2015–2020
University of Huelva	1	8	18	21	22
University of Basque Country		4	5	2	
University of Granada					9
Others				4	9
In total: 103	1	12	23	27	40
